# Early General Hypothermia Improves Motor Function after Spinal Cord Injury in Rats; a Systematic Review and Meta-Analysis

**Published:** 2020-10-06

**Authors:** Mahmoud Yousefifard, Mohammad Hossein Vazirizadeh-Mahabadi, Leila Haghani, Farhad Shokraneh, Alexander R. Vaccaro, Vafa Rahimi-Movaghar, Mostafa Hosseini

**Affiliations:** 1 Physiology Research Center, Iran University of Medical Sciences, Tehran, Iran.; 2 Student Research Committee, Faculty of Medicine, Iran University of Medical Sciences, Tehran, Iran.; 3 School of Medicine, International Campus, Tehran University of Medical Science, Tehran, Iran.; 4 Cochrane Schizophrenia Group, Institute of Mental Health, University of Nottingham, Nottingham, UK.; 5 Department of Orthopedics and Neurosurgery, Rothman Institute, Thomas Jefferson University, Philadelphia, USA.; 6 Sina Trauma and Surgery Research Center, Tehran University of Medical Sciences, Tehran, Iran.; 7 Brain and Spinal Injuries Research Center (BASIR), Neuroscience Institute, Imam Khomeini Hospital, Tehran University of Medical Sciences, Tehran, Iran.; 8 Pediatric Chronic Kidney Disease Research Center, Tehran University of Medical Sciences, Tehran, Iran.; 9 Department of Epidemiology and Biostatistics, School of Public Health, Tehran University of Medical Sciences, Tehran, Iran.

**Keywords:** Spinal Cord Injuries, Hypothermia, Movement Disorders, Rats

## Abstract

**Introduction:**

There is still controversy about the effect of early hypothermia on the outcome of spinal cord injury (SCI). The aim of this review article is to investigate the effect of local or general hypothermia on improving the locomotion after traumatic SCI.

**Methods:**

Electronic databases (Medline and Embase) were searched from inception until May 7, 2018. Two independent reviewers screened and summarized the relevant experimental studies on hypothermia efficacy in traumatic SCI. The data were analyzed and the findings were presented as pooled standardized mean difference (SMD) and 95% confidence interval (95% CI).

**Results:**

20 papers containing 30 separate experiments were included in meta-analysis. The onset of hypothermia varied between 0 and 240 minutes after SCI. Administration of hypothermia has a positive effect on locomotion following SCI (SMD=0.56 95% CI: 0.18-0.95, p=0.004). Subgroup analysis showed that general hypothermia improves locomotion recovery (SMD =0.89, 95% CI: 0.42 to 1.36; p <0.0001), while local hypothermia does not have a significant effect on motor recovery (SMD=0.20, 95 % CI: -0.36-0.76, p=0.478). In addition, general hypothermia was found to affect motor recovery only if its duration was between 2 and 8 hours (SMD=0.89; p<0.0001) and the target temperature for induction of hypothermia was between 32 and 35° C (SMD=0.83; p<0.0001).

**Conclusion:**

We found that general hypothermia improves locomotion after SCI in rats. Duration of induction and the target temperature are two essential considerations for general therapeutic hypothermia.

## Introduction

Current treatments that are considered to improve spinal cord injury (SCI) outcome include medicinal therapy (e.g. methylprednisolone), surgery, and rehabilitation (1-3). However, these clinical managements in SCI have not been satisfactory and even some recent studies recommended against these managements, such as application of methylprednisolone 8 hours post-SCI (2). While researchers are looking for new therapeutic interventions such as stem cell and laser therapy to postpone or stop the pathological process of SCI (4-10), hypothermia is one of the old therapeutic interventions that has been suggested in different studies. The advantages of reducing body temperature have been reported in different subjects such as cardiac arrest (11), neonatal ischemic-hypoxic encephalopathy (12, 13), hepatic encephalopathy (14), cerebral aneurysm (15), stroke (16-18), traumatic brain injury (19, 20) and SCI (21-23). For instance, in a clinical trial, Kim et al. demonstrated that prehospital induction of mild hypothermia improved survival and neurological status of cardiac arrest patients (11). In another study, Seo et al. reported that hypothermia has a neuroprotective effect and it could decrease apoptosis and autophagy after SCI (22). 

Hypothermia can be classified into three groups, including severe hypothermia (below 28°C), moderate hypothermia (28 to 32°C), and mild hypothermia (33-35°C). Early studies have shown the efficacy of local hypothermia in improving neurological complications in animal models (24-27), and the effectiveness of general hypothermia, reported in subsequent studies, was far greater (28). Human studies also revealed that hypothermia reduced the complications of SCI (29-31). However, there is still substantial controversy on the effectiveness of hypothermia in controlling post-SCI complications. Some other studies failed to find a similar effect. For example, Lo et al. have demonstrated that using general hypothermia only improved locomotion within the first weeks after SCI, but ultimately, after 8 weeks of follow-up, the motor score did not differ from the control group (32). In another study, Maybhate et al. showed that general hypothermia provided a positive neuroprotective effect in acute and subacute phases of SCI and could improve hind limb locomotion (33). However, Batchelor et al. (34) and Morizane et al. (35) showed that hypothermia provided significant improvement in locomotion 8 weeks after SCI. In addition, whether the intensity of hypothermia, lesion site, the severity of injury, and other factors influence the effect of therapeutic hypothermia on improvement of SCI outcome remains widely unknown. Batchelor et al. (34) showed that hypothermia could improve locomotion if the duration of hypothermia induction was 8 hours. However, Lo et al. did not show a significantly higher post-injury locomotion after 6-hour hypothermia (36). Teh et al. (37) showed that moderate hypothermia did not have a significant effect on motor function after SCI, while Morizane et al. (35) study showed the contrary. Therefore, in the present study, we aimed to systematically analyze the effect of hypothermia on improving locomotion recovery in animal models of SCI.

## Methods

**Study design and search strategy**

The method of searching databases and performing analyses was the same as the previously published Meta-analyzes by the present researchers (38-43). Following the selection of keywords related to SCI and hypothermia, Medline and Embase databases were searched from inception until May 7, 2018. To find additional articles, bibliography of related article and reviews was screened. Google search engine, Google Scholar and the ProQuest were also searched. The search query for Medline (via OvidSp) is provided below.

1. Spinal Cord Injury / OR Quadriplegia / OR Paraplegia / OR (Spinal Cord / AND "Wounds and Injuries") OR (("Spinal Cord" adj (Injur * OR Contus * OR Trauma * OR Posttrauma * OR Transect * OR Lacerat * OR Compromi * OR Lesion * OR Rupture *)) OR Quadripleg * OR Paraplegic * OR Tetraplegi * OR Quadripares' s OR ((Trauma * OR Posttrauma *) adj Myelopath *)) ti.ab.

2. Cold Temperature / OR Hypothermia / OR Ex Cryotherapy / OR Cryoanesthesia / OR Ex Hypothermia, Induced / OR Body Temperature Changes / OR Gastric Hypothermia / OR (Cryoan? Esthe * OR Cryogen * OR Cryotherap * OR Cryotherm * OR Cryotreat * OR Cold * OR Cool * OR Chill * OR Hypotherm * OR (Temperature adj4 (Decreas * OR Reduc * OR Low * OR Minim * OR Taper *)) OR ((Decreas * OR Reduc * OR Low * OR Minim * OR Taper *) adj4 Temperature) OR "Artificial Hybernation" OR (Refrigerat * adj An? Esthe *)) ti.ab.

3. 1 AND 2

**Eligibility criteria**

All rat studies that examined the effects of general or local hypothermia on locomotion recovery following traumatic SCI (contusion, compression, hemisection, transection, crush injury model) were included. Exclusion criteria consisted of in vitro studies, lack of functional assessment, lack of control group, non-traumatic SCI (aortic cross-clamping model of SCI), mild severity of SCI model, combination therapy of hypothermia with other treatments, not reporting details of hypothermia administration and reviews.

**Quality assessment and Data Extraction**

Two independent reviewers initially screened titles and abstracts, identified potentially relevant articles, and screened their full texts based on inclusion and exclusion criteria. They independently recorded animals’ age/weight, strain, species and sex, mechanism of the induction of SCI, details of hypothermia induction, number of samples and locomotion score. In animal studies, examination for locomotion recovery is usually conducted in several time sessions, so we only included the last session of follow-up. If the results were presented in the charts, data were extracted using the WebPlotDigitizer software. WebPlotDigitizer is a reliable software for extracting data and its accuracy has been proven in a previous study (44). Severity of injury was categorized based on the definition given in the article by Cheriyan et al. (45). Hypothermia was classified into three groups, including severe hypothermia (target temperature below 28°C), moderate hypothermia (target temperature between 28 and 32°C), and mild hypothermia (target temperature between 33 and 35 °C). Any disagreements were resolved through discussion with the third reviewer.

Qualitative assessment of papers was performed based on the method suggested in Hassannejad et al. study (46). Disagreements were resolved through discussion with the third reviewer.  

**Statistics**

Data were analyzed using STATA software version 14.0. Locomotion score was recorded as mean and standard deviation and using the "metan" command, a random-effect analysis was performed. Finally, the output was presented as pooled standardized mean difference (SMD) and 95% confidence interval (95% CI). I^2^ tests were used to evaluate heterogeneity between the studies. In cases with high levels of heterogeneity (I^2^≥ 50%), subgroup analysis was performed to determine the cause of heterogeneity. Funnel Plot was used to identify publication bias using Egger's test (47).

## Results

**Studies’ characteristics**

**Figure 1:**
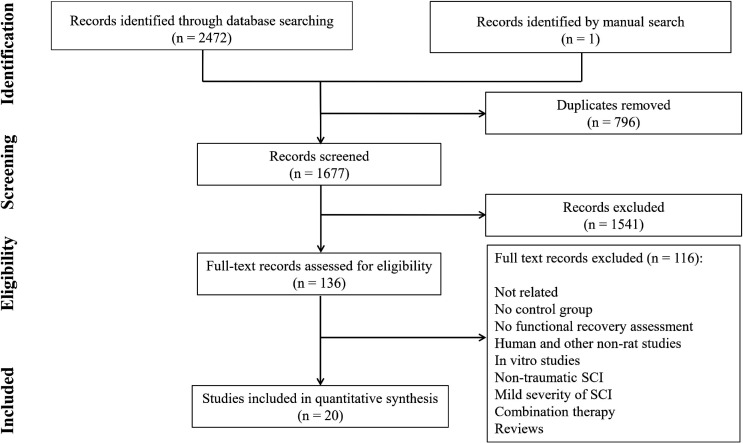
PRISMA flow diagram of the present meta-analysis

The titles and abstracts of 1677 non-duplicate articles were screened and then full text of 136 articles were selected for in-depth assessment. Finally, the data of the 20 included studies were pooled in a meta-analysis (33-37, 48-62) (Fig. 1). These studies contained 30 separate experiments. All studies used the compression / contusion model to induce spinal cord injury. Intensity of injury was moderate in 22 experiments and severe in eight experiments. Location of injury was cervical in two experiments, thoracic in 26 experiments, and in the thoracolumbar or lumbar regions in two experiments. The onset of hypothermia varied between 0 and 240 minutes after SCI (0 minutes in 11 experiments and 30 minutes in nine experiments). The duration of hypothermia also varied between 60 minutes and 2880 minutes. 15 experiments assessed the effect of local hypothermia and 15 experiments investigated the effect of general hypothermia on locomotion recovery. Table 1 shows a summary of the eligible articles.

**Quality assessment of articles and publication bias**

**Figure 2:**
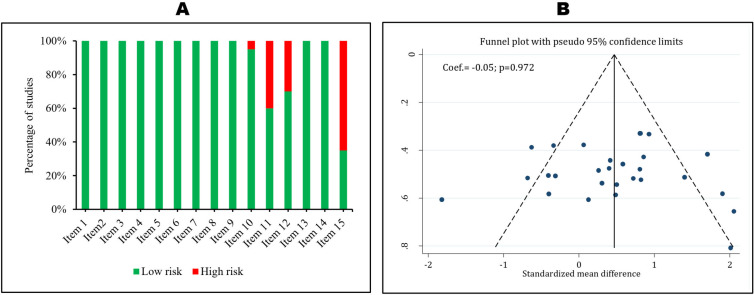
Quality assessment (A) and risk of publication bias (B) in the current meta-analysis. A) Item 1. Species; item 2. Using appropriate tests; item 3. Severity of injury; item 4. Level of injury; item 5. Age/weight; item 6. Number of animals per group; item 7. Designation of strain; item 8. Definition of control; item 9. Description of statistical analysis; item 10. Regulation and ethics; item 11. Bladder expression; item 12. Blindness of assessor; item 13. Genetic background; item 14. Method of allocation to treatments; item 15. Description of the reasons for excluding animals from the experiment during the study. B) There is no publication bias in the present meta-analysis

Table 2 and Fig. 2 show the quality status and risk of bias among the studies. There was no publication bias in present meta-analysis (p=0.972). Among the studies, 40.0% did not report bladder expansion and 65.0% did not describe the reasons for excluding animals from the experiment. There was one study that did not have regulation and ethical statement, and six studies that did not clearly report blinding status. 

**The effect of hypothermia on locomotion recovery**

**Figure 3:**
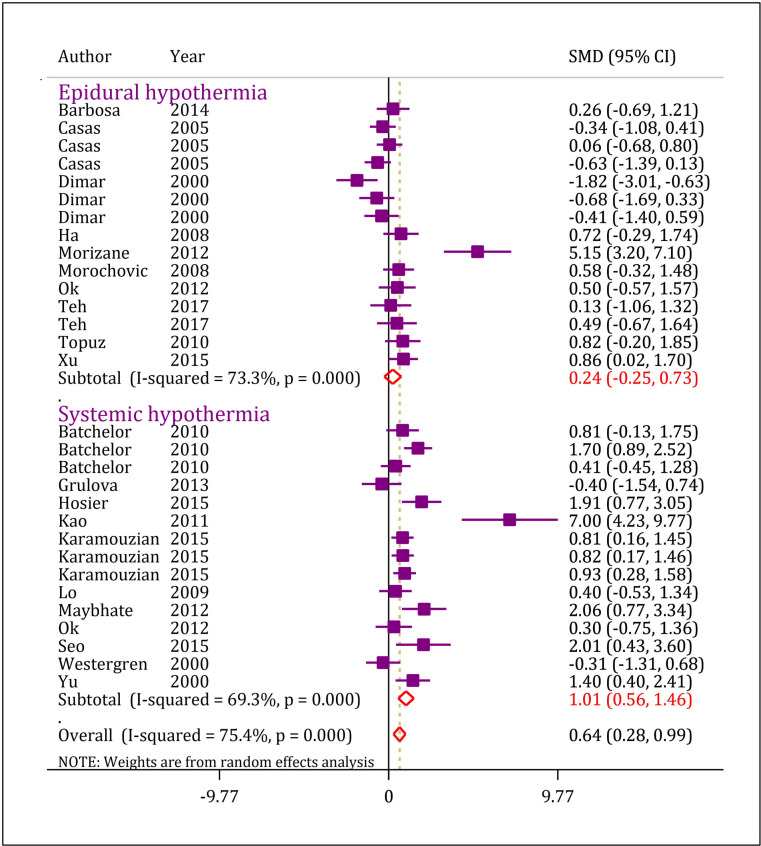
Forest plot of general and local hypothermia on motor function recovery after spinal cord injury. Data were extracted from 20 studies including 30 separate experiments. CI: Confidence interval; SMD: Standardized mean difference

The findings of this meta-analysis showed that hypothermia (local and general) has a positive effect on locomotion following SCI (Fig. 3) (SMD = 0.64, 95% CI: 0.28 to 0.99, p < 0.0001, I^2^ = 75.4%, p <0.0001). Subgroup analysis showed that general hypothermia improves locomotion (SMD = 1.01, 95% CI: 0.56 to 1.46; p <0.0001), while local hypothermia does not have any effect on motor recovery (SMD = 0.24, 95% CI: -0.25 to 0.73; p = 0.341). 

**Subgroup analysis of general hypothermia on SCI**

The analysis showed that general hypothermia only improves locomotion in moderate injuries (SMD = 1.00, p < 0.0001), whereas in severe injuries, does not have a significant effect (SMD = 1.09; p = 0.173). Level of injury was another factor affecting the influence of general hypothermia. The effect of general hypothermia on locomotion recovery was significant only in case of thoracic injuries (SMD = 1.00; p < 0.0001). In addition, it was found that general hypothermia could only improve locomotion if its duration was between 2 and 8 hours (SMD = 0.93; p < 0.0001), but when the duration was less than 2 hours (p = 0.102) or more than 8 hours (p = 0.572), its effect was not significant.

Another factor influencing the effectiveness of general hypothermia was the intensity of hypothermia. Analysis demonstrated that induction of mild general hypothermia (SMD = 0.95; p <0.0001) resulted in motor recovery improvement, while moderate (p = 0.380) and severe (p = 0.571) general hypothermia had no effect on locomotion recovery. Another factor influencing locomotion recovery was the duration of follow-up. Analyses showed that the role of general hypothermia in improving locomotion recovery is significant only when the animals were followed up for at least 4 weeks (SMD _for 4 to 7 weeks follow-up _= 1.00, p < 0.0001; SMD _for 8 weeks and more follow-up _= 0.85, p = 0.003) (Table 3).

**Table 1:** Characteristics of included animal studies

**Author; Year; Country**	**Sample size (control; treated)**	**Gender; Strain; Species**	**Injury model**	**Severity**	**Injury level**	**Onset of hypothermia post-injury (min)**	**Hypothermia duration (min)**	**Type of hypothermia**	**Cooling temperature (°C)**	**Follow up duration (day)**
Barbosa; 2014; Brazil	15; 15	Male and female; Wistar; Rat	Contusion	Moderate	T9-T10	0	20	Local	25	42
Batchelor; 2010; Australia	36; 36	Female ; Fischer; Rat	Contusion	Moderate	T8	30	450	General	33	56
Casas; 2005; USA	14; 42	Female ; SD; Rat	Contusion	Moderate	T10	30	180	Local	24±2.3	42
Dimar; 2000; USA	26; 26	Male; SD; Rat	Contusion	Moderate	T10	0	120	Local	19	35
Grulova; 2013; Slovakia	12; 4	Male; Wistar; Rat	Compression	Moderate	T8–T9	0	198	General	32.0	28
Ha; 2008; Korea	8; 8	Male; SD; Rat	Contusion	Severe	T9	0	2880	Local	30	7
Hosier; 2015; USA	10; 8	Female ; Long-Evans; Rat	Contusion	Severe	C7	240	240	General	33.0±0.3	42
Kao; 2011; Taiwan	8; 8	Male; SD; Rat	Contusion	Moderate	T8-T9	0	120	General	31 to 35	4
Karamouzian; 2015; Iran	20; 60	Male; Wistar; Rat	Contusion	Moderate	T8–9	30	180	General	33.5±0.5	42
Lo; 2009; USA	9; 9	Female; Fischer; Rat	Contusion	Moderate	C5	5	240	General	33.0±0.3	56
Maybhate; 2012; USA	8; 7	Female; Lewis; Rat	Contusion	Moderate	T8	120	120	General	32	28
Morizane; 2012; Japan	10; 9	Female ; Wistar; Rat	Contusion	Severe	T11	0	2880	Local	33	56
Morochovic; 2008; Slovakia	10; 10	Male; SD; Rat	Compression	Severe	T8-T9	25	60	Local	28.5±0.3	28
Ok; 2012; Korea	7; 14	Male; SD; Rat	Contusion	Severe	T9	0	2880	Local and general	20	42
Seo; 2015; Korea	5; 5	Male; SD; Rat	Contusion	Moderate	T9	15	240	Systemic	30-32	42
Teh; 2017; Singapore	6; 11	NR; SD; Rat	Contusion	Moderate	T8	120	300	Local	32±0.5	42
Topuz; 2010; Turky	8; 8	Male; Wistar; Rat	Compression	Moderate	T10-T12	30	120	Local	27-29	42
Westergren; 2000; Sweden	9; 7	Male; SD; Rat	Compression	Moderate	T7-T8	60	120	General	30	14
Xu; 2016; China	12; 12	Male; SD; Rat	Compression	Moderate	T10	0	160	Local	18	21
Yu; 2000; USA	8; 12	Female ; SD; Rat	Contusion	Moderate	T10	30	240	General	33.1	44

GMLC: GottingenMinnesotaLiběchov crossbred; SD: Sprague–Dawley

**Table 2:** Quality assessment of included studies

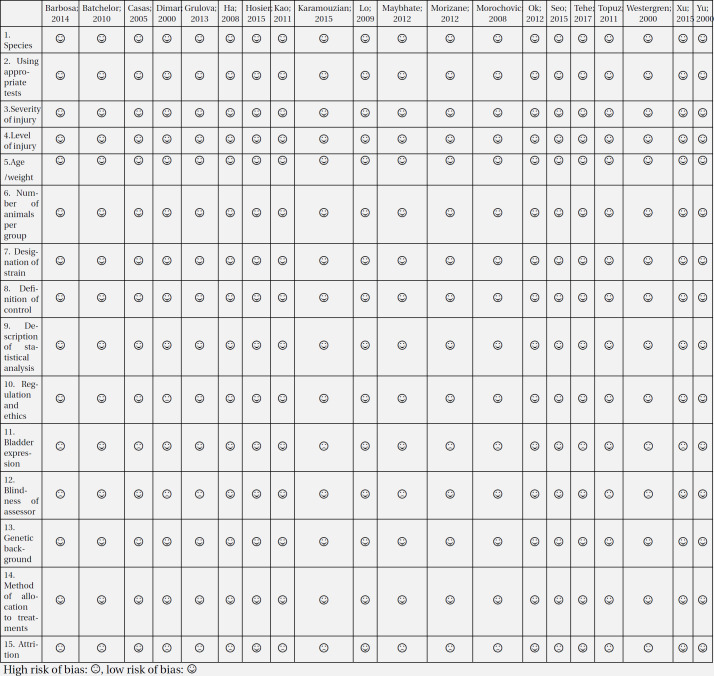

**Table 3:** Subgroup analysis for assessment of general hypothermia on motor function recovery

**Subgroups**	**Number of experiments**	**Effect size**		**Heterogeneity (p value)**
		**SMD (95% CI)**	**p value**	
Severity of injury*				
Moderate	13	1.00 (0.51 to 1.49)	<0.001	71.0% (<0.0001)
Severe	2	1.09 (-0.48 to 2.66)	0.173	75.5% (0.043)
**Level of injury**				
Cervical	2	1.12 (-0.36 to 2.59)	0.137	75.0% (0.046)
Thoracic	13	1.00 (0.51 to 1.49)	<0.001	71.1% (<0.0001)
**Onset of hypothermia after SCI**				
1 hour or less	11	0.92 (0.31 to 1.52)	0.003	74.3% (<0.0001)
More than 1 hour	4	1.22 (0.66 to 1.78)	<0.0001	40.9% (0.166)
**Duration of hypothermia **				
2 hours or less	3	2.64 (-0.52 to 5.80)	0.102	92.7% (<0.0001)
2 to 8 hours	11	0.93 (0.58 to 1.23)	<0.0001	41.2% (0.074)
More than 8 hours	1	0.30 (-0.75 to 1.36)	0.572	0.0% (>0.999)
**Intensity of hypothermia**				
Mild (32 to 35 °C)	12	0.95 (0.60 to 1.31)	<0.0001	45.0% (0.052)
Moderate (28 to 31.9 °C)	2	2.66 (-0.76 to 6.09)	0.380	92.4% (<0.0001)
Severe (less than 28 °C)	1	0.30 (-0.75 to 1.36)	0.571	0.0% (>0.999)
**Follow up duration**				
Less than 4 weeks	2	3.22 (-3.94 to 10.38)	0.377	95.8% (<0.0001)
4 to 7 weeks	9	1.00 (0.57 to 1.43)	<0.0001	47.4% (0.055)
8 weeks and more	4	0.85 (0.22 to 1.48)	0.008	50.4% (0.109)

*, Severity of injury was categorized based on the definition given in the article by Cheriyan et al (45).

CI: Confidence interval; SMD: Standardized mean difference; SCI: Spinal cord injury

**Table S1:** The conflict of interest statement of Prof. Alex R. Vaccaro. Health care entity relationships and investments

***Entity***	***Relationship (see legend below)***
Replication Medica	d
Medtronics	c
Stryker Spine	c,
Globus	c,d
Paradigm Spine	d
Stout Medical	d
Progressive Spinal Technologies	d
Advanced Spinal Intellectual Properties	d
Aesculap	c
Spine Medica	d
Computational Biodynamics	d
Spinology	d
Flagship Surgical	d
Cytonics	d
Bonovo Orthopaedics	d
Electrocore	d
Insight Therapeutics	d
FlowPharma	d
Rothman Institute and Related Properties	d
AO Spine	g
Innovative Surgical Design	d
Orthobullets	d
Thieme	c
Jaypee	c
Elseviere	c
Taylor Francis/Hodder and Stoughton	c
Expert testimony	g
Vertiflex	d
Avaz Surgical	d
Dimension Orthotics, LLC	d
SpineWave	c
Atlas Spine	c
Nuvasive	d
Parvizi Surgical Innovation	d
Franklin Bioscience	d
Deep Health	d

a. Consulting / Independent Contractor

b. Service on Scientific Advisory Board / Board of Directors / Service on Committees

c. Receipt of Royalty Payments

d. Stock / Stock Option Ownership Interests

e. Institutional / Educational Grant

f. Deputy editor/ Editor/Editorial Board

g. Member in good standing// Independent Contractor

## Discussion

The present study showed that general hypothermia has a positive effect on locomotion recovery following SCI, but local hypothermia did not have any effect on locomotion recovery. General hypothermia's effectiveness in improving motor recovery can be attributed to its angiogenic, neurogenic, and anti-inflammatory effects (53). Following general hypothermia, microglial proliferation, TNF-α production, and neutrophil migration are significantly decreased (53, 63). However, local hypothermia reduces the survival of the grey matter in the spinal cord (48). Direct cold contact decreases perfusion of the injured spinal cord (48, 64), which increases cell mortality and cannot have protective effects.

In a meta-analysis, Batchelor et al. (2013) have demonstrated that general and local hypothermia can improve animal locomotion by about 24.5% and 26.2%, respectively, (28). However, the present study shows that local hypothermia does not have an effect on locomotion recovery after SCI. The reason for this discrepancy could be the inclusion of a variety of species, such as primates, in Batchelor's systematic review (Batchelor review). In addition, details of effect size calculation, the included articles, and quantitative control of the eligible studies were not provided in that review. Moreover, the results of neurological assessments were pooled with locomotion scores, which may result in considerable heterogeneity and possible bias. In Batchelor review, when the analysis was limited to locomotion (BBB test), it was found that the effectiveness of local hypothermia was only 8.8% (95% CI: 0.06 to 16.7%). Therefore, in that review, local hypothermia has had limited effect on post-SCI locomotion recovery. In 2016, Alkabie et al. performed a systematic review on articles from the Medline database, and evaluated the role of hypothermia on traumatic SCI outcome in animal studies. Their findings showed that hypothermia could improve locomotion recovery in animals. However, only searching in MEDLINE and not performing a meta-analysis and subgroup analysis were the greatest weaknesses of the study (65).

One of the most important findings of the present study is the effectiveness of mild general hypothermia in improving locomotion recovery. This finding is completely in discrepancy with the Batchelor review, which showed that the highest efficacy was observed when hypothermia was induced at 4 to 19°C. The reason for this controversy can be the difference in the type of analyses. Analyses of Batchelor et al. were limited to the local hypothermia (28), while we also performed analysis on general hypothermia. The results of a systematic review on human studies (level IV of evidence) was consistent with our findings, showing that mild general hypothermia is a safe method with improved outcomes of SCI (66). It seems that in severe and moderate general hypothermia, the spinal cord blood flow is reduced, with more destructive, rather than protective, effects leading to an increase in death of residual spinal cord cells (67).

Treatment duration was another factor influencing the effectiveness of general hypothermia. Our findings showed that if the treatment duration was less than 2 hours or more than 8 hours, general hypothermia had no effect on the outcome of SCI, which perhaps suggested a role of a therapeutic window in the effectiveness of an intervention. It is likely that less than 2 hours of induction of hypothermia is not sufficient for the researchers to observe its effects on locomotion recovery, whereas treatment for more than 8 hours may increase the destructive effects of hypothermia on motor function.

One of the limitations of the present study was the existence of significant heterogeneity between studies. Although subgroup analysis was performed, in some cases the source of heterogeneity was not recognized. Also, the duration of follow-up varied between studies, which could have had affected the findings. Subgroup analysis showed that the role of hypothermia in recovery was significant only when the animals were followed for at least 4 weeks.

## Conclusion

We found that general hypothermia improves locomotion after SCI in rat models. Duration of general hypothermia being between 2 and 8 hours, and hypothermia being mild (the target temperature being 32 to 35°C) are two essential considerations for best results in general therapeutic hypothermia. 
